# Structure of a conserved hypothetical protein SA1388 from *S. aureus *reveals a capped hexameric toroid with two PII domain lids and a dinuclear metal center

**DOI:** 10.1186/1472-6807-6-27

**Published:** 2006-12-23

**Authors:** Kumar Singh Saikatendu, Xuejun Zhang, Lisa Kinch, Matthew Leybourne, Nick V Grishin, Hong Zhang

**Affiliations:** 1 Department of Biochemistry, University of Texas Southwestern Medical Center, 5323 Harry Hines Blvd, Dallas TX 75390-8816, USA; 2 Howard Hughes Medical Institute, University of Texas Southwestern Medical Center, 6001 Forest Park Blvd., Dallas, Texas 75390-9050, USA; 3 Department of Geoscience, University of Texas at Dallas, Richardson, Texas 75083-0688, USA; 4 Department of Cell Biology, The Scripps Research Institute, 10550 N. Torrey Pines Rd, La Jolla, CA, 92037, USA

## Abstract

**Background:**

The protein encoded by the SA1388 gene from *Staphylococcus aureus *was chosen for structure determination to elucidate its domain organization and confirm our earlier remote homology based prediction that it housed a nitrogen regulatory PII protein-like domain. SA1388 was predicted to contain a central PII-like domain and two flanking regions, which together belong to the NIF3-like protein family. Proteins like SA1388 remain a poorly studied group and their structural characterization could guide future investigations aimed at understanding their function.

**Results:**

The structure of SA1388 has been solved to 2.0Å resolution by single wavelength anomalous dispersion phasing method using selenium anomalous signals. It reveals a canonical NIF3-like fold containing two domains with a PII-like domain inserted in the middle of the polypeptide. The N and C terminal halves of the NIF3-like domains are involved in dimerization, while the PII domain forms trimeric contacts with symmetry related monomers. Overall, the NIF3-like domains of SA1388 are organized as a hexameric toroid similar to its homologs, *E. coli *ybgI and the hypothetical protein SP1609 from *Streptococcus pneumoniae*. The openings on either side of the toroid are partially covered by trimeric "lids" formed by the PII domains. The junction of the two NIF3 domains has two zinc ions bound at what appears to be a histidine rich active site. A well-defined electron density corresponding to an endogenously bound ligand of unknown identity is observed in close proximity to the metal site.

**Conclusion:**

SA1388 is the third member of the NIF3-like family of proteins to be structurally characterized, the other two also being hypothetical proteins of unknown function. The structure of SA1388 confirms our earlier prediction that the inserted domain that separates the two NIF3 domains adopts a PII-like fold and reveals an overall capped toroidal arrangement for the protein hexamer. The six PII-like domains form two trimeric "lids" that cap the central cavity of the toroid on either side and provide only small openings to allow regulated entry of small molecules into the occluded chamber. The presence of the electron density of the bound ligand may provide important clues on the likely function of NIF3-like proteins.

## Background

Despite the increased sophistication of annotation tools, a significant number of protein sequences emerging from genome sequencing efforts continue to remain in the realm of "hypothetical proteins" with little or no functional annotation associated with them. Ultimately, a definite functional annotation would require experimental characterization, which is often time consuming and expensive. Careful remote homology detection and manual analysis has in many occasions helped to glean useful structural and functional insights into these so-called "hypothetical proteins". Typically, such studies involve a combination of profile based methods like transitive PSI-BLAST [1], COMPASS [2] and HMMer [3] as well as structure prediction and fold recognition methods [4-6].

One such study [7] had analyzed sequences of the ubiquitously found protein modules homologous to the nitrogen regulatory PII proteins as defined in the COG [8] and Pfam databases [9]. This comprehensive analysis expanded the PII protein superfamily to include five very divergent groups of proteins, with below random (~1%) pairwise sequence identities between some members of distant groups. Nevertheless, each group has distinct patches of conserved local similarities and was predicted to retain the same overall structural fold as PII and a trimeric structure essential for ligand-binding site formation. The PII-like proteins are small protein modules of ferredoxin-like fold containing a core (βαβ)_2 _secondary structural pattern, and function as trimers. While the nitrogen regulatory PII proteins that belong to the Group II of the superfamily have been studied extensively [10], the functions of other groups in the superfamily are either poorly understood or completely unknown. One group of PII-like proteins, Group III, is significantly larger (~370 aa) than a typical PII protein domain (~112 aa). In these proteins, the PII domain is embedded within the central region of the polypeptide while the N- and C-terminal regions together belong to the NIF3 (NGG1p interacting factor 3)-like protein family. Presumably, the PII domain of these proteins would play some sort of ligand binding and signalling role analogous to that of classical PII proteins [10,11], while the function of the NIF3-like domains is not known.

We chose the Group III proteins of the PII superfamily (represented by the *Staphylococcus aureus *protein SA1388) for structure determination with two primary objectives: to structurally characterize both the Group III PII-like domain and NIF3 domains which may provide clues to their potential function that is otherwise unattainable from sequence information alone. Apart from providing confirmation of our prediction of the central P-II domain, the structure of SA1388 would also be important for functional analysis of the NIF3 domains, which were recently highlighted in the top 10 list of important structural targets due to their broad phylogenetic distribution, sequence conservation patterns with putative "*active-site like*" features and their uncharacterized role as a putative regulatory molecules of eukaryotic transcription [12].

## Results

### Description of SA1388 monomer

**Figure 1:**
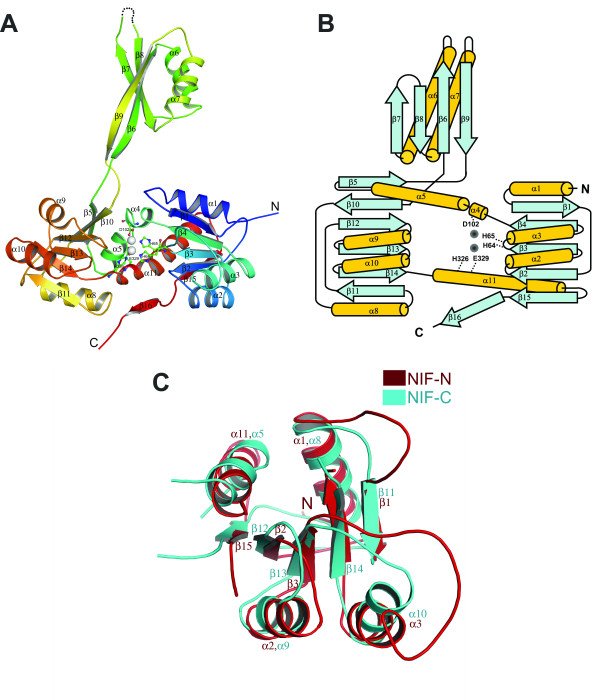
**Structure and topology of SA1388**. (**A**) Ribbon diagram showing the overall fold and three-domain organization of SA1388 monomer. Secondary structures are colored from blue (N-terminus) to red (C-terminus). Helices are labelled from α1-α11 while strands are labelled β1-β16. The two bound zinc atoms are shown as grey balls and the liganding residues are shown in ball-and-stick representation. (**B**) Topology diagram showing the connectivity of the secondary structures. Helices are colored yellow and the strands are in cyan with the same numbering as in 1A. (**C**) Superposition of the N (Red) and C terminal (blue) NIF3 domains on each other. The secondary structures are numbered as in Figure 1A.

The final model of SA1388 (gi:54040095 Swissprot: P67273;) refined to 2.0Å resolution consists of two subunits in the asymmetric unit. One subunit is traceable across the entire length of polypeptide from the N- to the C-terminus (residues 1 to 366) with the exception of a loop region containing 25 residues (168–193) in the central PII domain. In the other monomer, several more residues are disordered (139 – 193). A ribbon representation of one SA1388 monomers is shown in Figure 1A.

Overall, the protein is organized into three distinct structural domains with interconnecting topological connectivities (Figure 1B). The two NIF3 domains at the N- and C-terminus of the protein (henceforth denoted as NIF3-N and NIF3-C, respectively) have the same overall fold as canonical NIF3-like proteins [13,14], with a central 5 stranded mixed β-sheet and a pair of helices on either side. NIF3-N and NIF3-C are structurally similar and are clearly superimposable as shown in Figure 1C, and therefore are probably a result of gene duplication.

The middle region of the polypeptide (residues 126–236) bulges out between the two NIF3 domains and is structured as a classical PII-like fold with a (β/α/β)_2 _architecture (Figure 1A), confirming our earlier prediction [7]. This domain is relatively well ordered only in one of the two monomers in the asymmetric unit. In this monomer, although all the secondary structural elements are well defined, we do not observe electron density for residues 169–193 that connects the strands β7 and β8. In the other monomer, in addition to this loop, helix α6 and strand β7 are also disordered. Residues in this domain have substantially higher B factors, indicating a higher mobility compared to the rest of the protein.

### Structure comparison with other NIF3 and PII proteins

**Figure 2:**
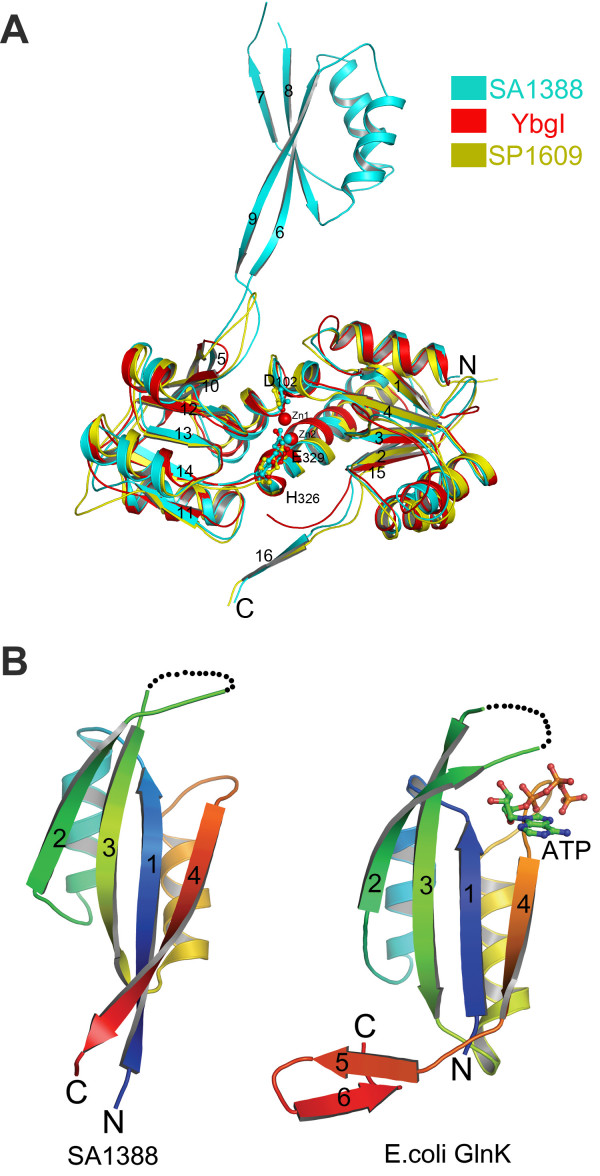
**Structure comparison of SA1388**. (**A**) Superimposed structures of SA1388 (shown in cyan) with two homologs, *E. coli *ybgI (PDB id: 1nmo; colored red) and SP1609 from *Streptococcus pneumoniae *(PDB id: 2fyw; colored yellow). The two bound Zn atoms of SA1388 are shown as spheres and superimposed on the two equivalent Fe atoms of ybgI. For clarity, only the strands of SA1388 are numbered using the same scheme as β1–16 of Figure 1A. Sidechains of three conserved residues that mediate metal binding in the three structures are shown in ball-and-stick representations with the numbering corresponding to that in SA1388. (**B**) Comparison of the PII domain of SA1388 (left) and a canonical PII domain seen in *E. coli *GlnK (PDB id: 2gnk) shown in a similar orientation. Colors are ramped from N terminus (blue) to C terminus (red) in both structures and the bound ATP of GlnK is shown in ball-and-stick representation. The disordered T-loop in the two structures is indicated by the dotted line.

Structure comparison of the entire polypeptide of SA1388 as well as the NIF3 domains using DALI [15] clearly identified two NIF3 homologous proteins, *E. coli *ybgI (PDB id: 2nmo; Z score = 25.5; RMSD of 1.9Å for 230 C_α _atoms) and SP1609 from *Streptococcus pneumoniae *(PDB id: 2fyw; Z score = 32.1; RMSD of 1.7 Å for 253 C_α _atoms). These two NIF3 homologs share sequence identities of 23% and 32%, respectively, with SA1388 sequence with the central PII-like domain (residues 128–235) excluded. A superimposition of the three structures is shown in Figure 2A. Both ybgI and SP1609 proteins have the same secondary structural connectivities as SA1388. However, unlike SA1388, these two proteins lack the middle PII-like domain.

The PII-like domain of SA1388 is topologically identical to several PII proteins with characterized structures (Figure 2B), such as GlnB [16] and GlnK [17], as well as PII-like protein CutA [18] and the C-terminal regulatory domain of ATP phosphoribosyltransferase (HisG) [19]. This domain has a core (βαβ)_2 _secondary structural pattern described in SCOP as a ferredoxin-like fold [20]. It remains to be determined whether the SA1388 PII domain binds ATP or other ligands either *in vivo *or *in vitro*.

### The NIF3 domains are involved in dimerization

**Figure 3:**
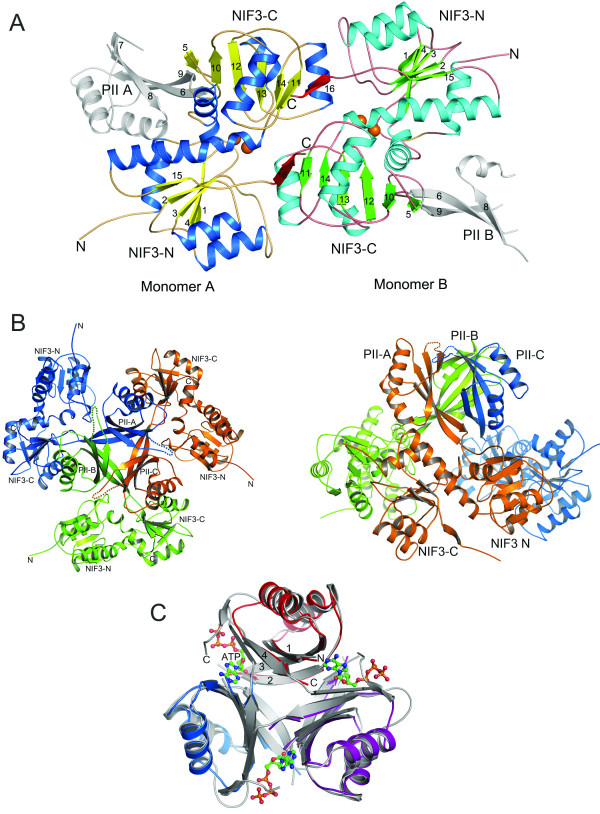
**Oligomeric assembly of SA1388**. (**A**) Schematic of an SA1388 dimer showing the dimer interface between the two NIF3 domains (NIF3-N and NIF3-C). The helices of monomer A are shown in blue and that of monomer B are in cyan. Similarly, the strands from monomer A are in yellow and that of monomer B in green. The strands are numbered according to the legend in Figure 1A. The two PII domains are shown in gray and the bound zinc atoms shown as orange balls. The domain-swapped β-strand (β16), that crosses over into the other subunit is highlighted in red. (**B**) Two orthogonal views of SA1388 trimers that make up half of the hexamer. The monomers are colored blue, green and orange respectively and the three domains are labelled. (**C**) Superimposed structures of SA1388 PII domain trimer (the three monomers are colored red, blue and violet, respectively) and that of *E. coli *glnK (PDB Id: 2GNK, colored gray). The four β strands in one SA1388 PII monomer are numbered according to the same scheme as in Figure 2B.

The two SA1388 monomers in the asymmetric unit tightly associate to form a dimer along the sides of the two NIF3 domains opposite to where the PII-domain is located. The two subunits are arranged in a head-to-tail manner with the NIF3-N of one subunit facing NIF3-C of the adjacent subunit, while the NIF3-C of the same subunit is nested in an elbow-like area between the two NIF3 domains of the second monomer. About 1828 Å^2^, or ~10% of the total surface area per monomer is buried in a largely hydrophobic (62%) interface (Figure 3A). There are also a few specific hydrogen bond interactions, including those between the side chains of Asp44-A and His308-B, Tyr80-A and Asp313-B, as well as that between the side chain of Asp305-A and main chain amide of His308. There is no salt bridge in the dimer interface. Additionally, the last β-strand (β16) of one monomer is part of the β-sheet of the other monomer (Figure 3A), with four main chain hydrogen bonds formed between the two adjacent β-strands from different monomers.

### The PII-like domains are involved in trimerization

The PII-like domain juts out of the dimer on the side opposite the dimer interface and forms homotrimers by interacting with the PII-like domains of symmetry related molecules along the three-fold axes (Figure 3B). The trimeric arrangement, where the individual β-sheets of each PII domain pack orthogonally is similar to that seen in several PII and PII-like proteins such as glnB [16], glnK [17], CutA [18] and the C terminal regulatory domain of HisG among others. A superimposition of PII trimer of SA1388 and that of ATP bound complex of *E. coli *glnK is shown in Figure 3C.

### SA1388 forms a capped toroidal hexamer

**Figure 4:**
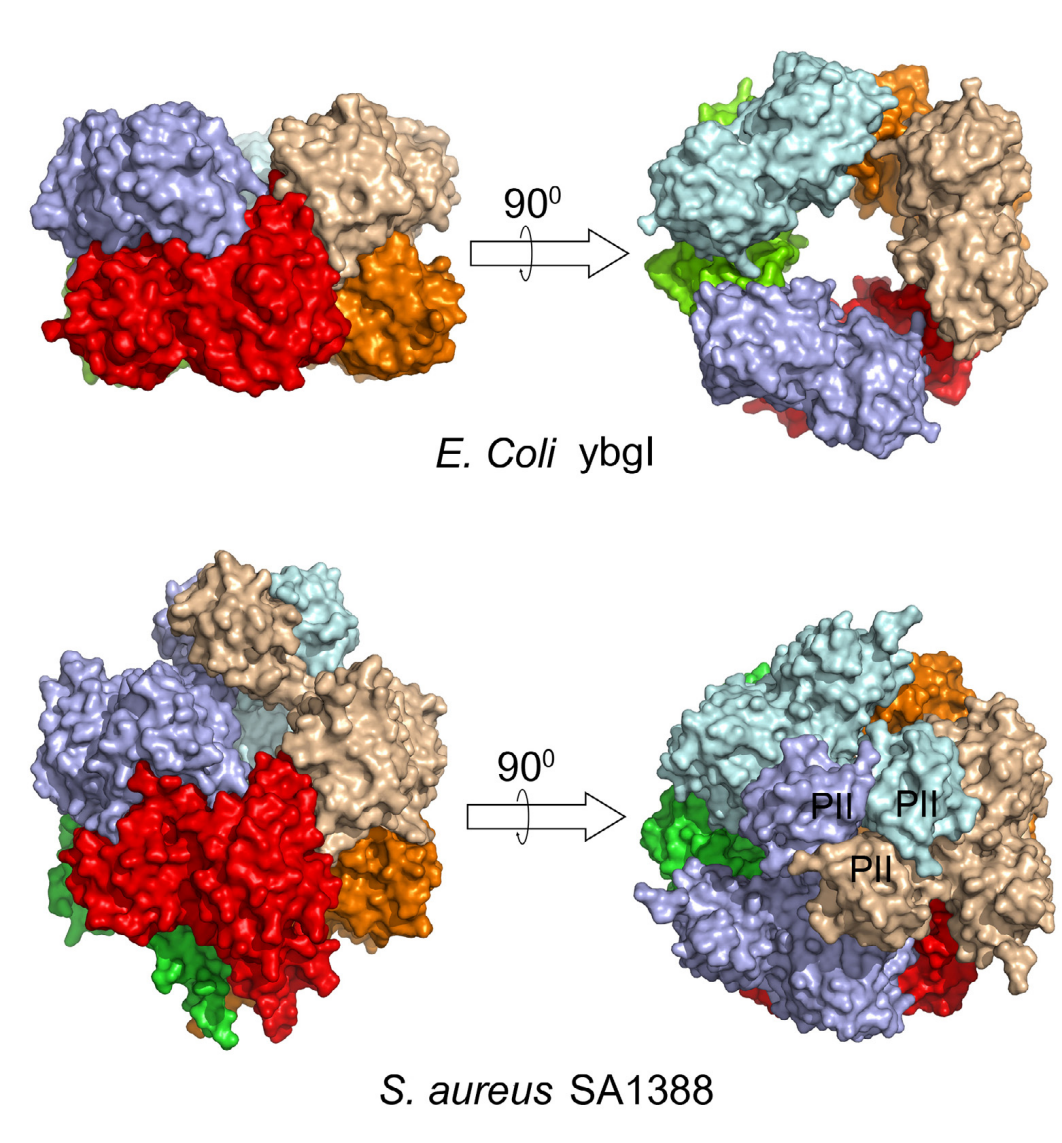
**Comparison of the hexameric toroids of *E. coli *ybgI and SA1388**. Surface diagrams showing two orientations of the *open *hexamer of *E. coli *ybgI (PDB id: 1nmo) (*upper panel*) and the partially closed hexamer of SA1388 (*lower panel*). Individual subunit surfaces are colored differently. The three PII domains that form the lid on either side of the toroid in SA1388 are indicated.

Further analysis of symmetry mates within the rhombohedral unit cell showed that the individual subunits are organized into hexamers with a cage-like appearance (Figure 4). The two NIF3 domains of each of the six monomers line the walls of this hollowed toroidal structure that has a central cavity of ~38Å diameter. The two NIF3 homologs, *E. coli *ybgI and SP1609 also assemble as hexamers in the crystals in a similar manner as that in SA1388, but their central cavity, which is of similar dimensions, is open at both ends. In SA1388, the two entries to the central hollow space are capped by the two PII domain trimers. However, this capping is not complete and leaves six smaller openings to the central cavity that are formed between the long stems connecting the PII-like domain and the NIF3 domains (Figure 4). The triangle shaped openings have approximate dimensions of ~20Å (length of each side of the triangle). These openings render the inside of the SA1388 hexamer solvent accessible and should allow access for small molecules to enter the putative active site (see below). Given the prevalent hexameric organization in all NIF3-like protein structures available so far, it is quite certain that the functional unit of these proteins is a hexamer.

### Description of the putative active site

**Figure 5:**
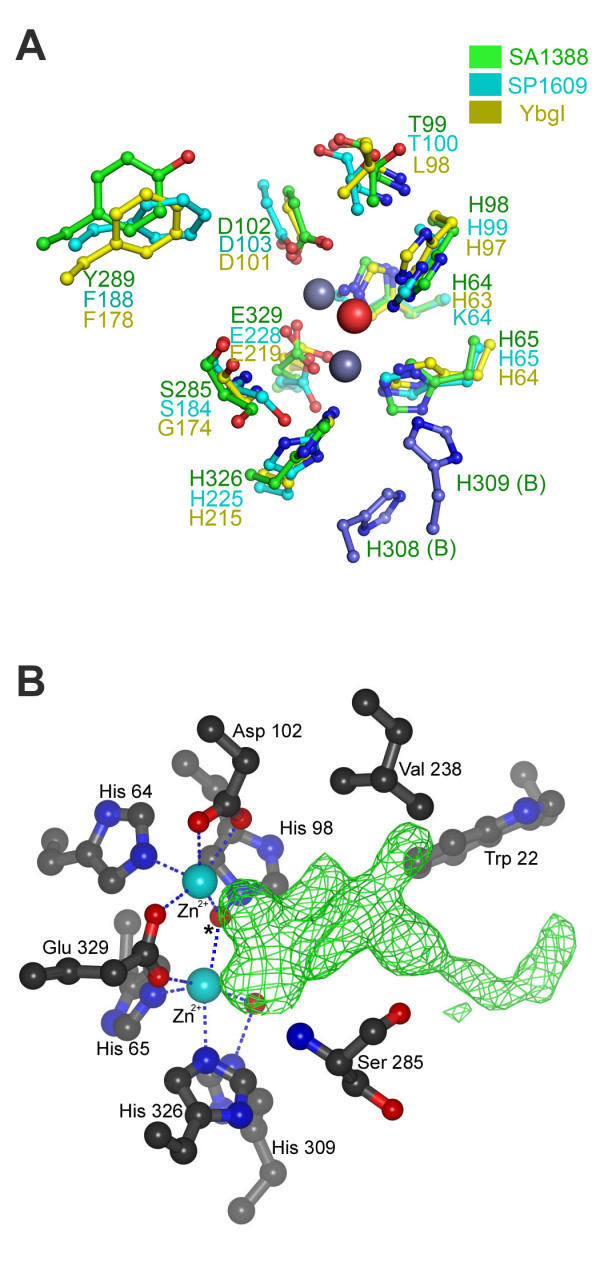
**The putative active site of SA1388**. (**A**) Conserved residues at the putative active sites of SA1388 (green), SP1609 (cyan) and *E. coli *ybgI (yellow). The two zinc ions of SA1388 and the bridging water molecule are shown as spheres and the equivalent residues in the three structures are labelled in their respective colors. The two histidine residues of SA1388 that are contributed by the adjacent subunit are colored in navy blue. (**B**) Electron density of the endogenous ligand (Fo-Fc map contoured at 3.0 *σ*) that is directly ligated to the active site zinc ions in SA1388. Residues interacting with the two zinc atoms are shown and the metal coordinating interactions are indicated by dotted lines. The bridging water molecule between the two Zn atoms is indicated by an asterisk (*).

We observe clear electron density for two tightly bound metal ions at the junction of the two NIF3 domains in each SA1388 monomer close to the protein surface that face inside the hexameric toroid (Figure 5A). The identity of the metal was determined to be zinc, first by an extended X-ray fluorescence scan at synchrotron and was further confirmed by Inductively Coupled Plasma (ICP) atomic emission spectrometry. We have therefore modelled two zinc atoms at the putative active site. Both metal atoms are tethered to the protein through several histidine and aspartate residues. Residue Glu329 is coordinated to both metal ions, while a water molecule, or more likely a hydroxide ion, also bridges the two metal ions. The configuration of this dinuclear metal center is very similar to that of the diiron site found in its *E. coli *homolog ybgI [14], and is reminiscent of the dinuclear mu-oxo diiron sites found in hemerythrins [21], ribonucleotide reductases [22] and purple acid phosphatase [23]. Some of the conserved residues at the metal center in the two structures along with that of SP1609 are shown in Figure 5A. The metal binding sites are remarkably similar in the three NIF3-like protein structures (*E. coli *ybgl, SP1609, and SA1388) and consist primarily of five histidines, one glutamate and one aspartate. These residues are among the most conserved residues in the NIF3-like protein superfamily.

Additionally, we observe a clear electron density for a ligand of unknown identity in close proximity to the two bound zinc atoms that would provide two additional metal ligands, one to each of the two metal ions to complete the octahedral metal center configuration (Figure 5B). By trial and error, we have ruled out all components in the crystallization solution that might have been inadvertently bound either during crystallization or during cryoprotection and therefore conclude that the observed ligand must in fact be an endogenously bound ligand that was co-purified after recombinant overexpression. The contours of the difference electron density map (Figure 5B) after final refinement suggests the presence of a head group directly ligated to the metal ions and a mostly aliphatic tail that has few specific polar interactions with the surrounding protein residues (Figure 5B). Two aromatic residues, a tyrosine (Y289, shown in Figure 5A) and a tryptophan (W22) lie in close proximity to the bound ligand and may be involved in ligand binding.

## Discussion

Although homologs of both PII and NIF3 proteins are found ubiquitously in all three kingdoms of life, the function of SA1388 and its homologs that contain both PII-like and NIF3-like domains remain completely unknown. The fusion of the two proteins in one peptide chain indicates a functional coupling of these two proteins. The structural features revealed in the present study, such as the cage-like hexameric toroid structure with its NIF3 domains as walls and the two PII-like domain trimers as lids, the dinuclear metal site, and the intrinsically bound ligand, may provide certain clues of its potential function. The primary role of the nitrogen regulatory PII proteins is to integrate various intracellular carbon and nitrogen signals by regulating enzymes involved in nitrogen assimilation [10]. PII exerts its regulatory effects by undergoing different post translational modifications, such as uridylylation [24] and phosphorylation [25] by various modifying enzymes in response to the primary cellular nitrogen signal glutamine. Structures of several PII and PII-like proteins have been solved including glnB (from *E. coli, T. thermophilus, Synecococcus *and *Herbaspirillum*), GlnA *(E. coli*), glnK (*E. coli*), HisG (*E. coli *and *M. tuberculosis*), CutA1 (human, rat and *T. maritima*), and a hypothetical protein from COG1993. Effectors of PII include transcription factors [10,11], signalling proteins e.g., histidine kinases [26], and metabolic enzymes like glutamine synthase [27]. Apart from its primary signalling modulator glutamine, PII proteins are known to bind a range of small molecule effectors such as ATP, UMP, and 2-ketoglutarate, which affects its function antagonistically to glutamine (reviewed in [10]). It is highly likely that the trimeric PII domain of SA1388 also plays a ligand induced signalling role and probably regulates the function of the NIF3-like domains.

Unlike PII domains, the NIF3-like domains have only recently begun to be structurally characterized, and their structure-function relationship remains sketchy. NIF3-like proteins are ubiquitously conserved from bacteria to higher eukaryotes [28]. They have been defined in uniprot database as the uncharacterized protein family UPF0135 that has 64 homologs [29]. Several homologs of this family (e.g., human and mouse) have a similar overall three-domain organization as in SA1388, while others (e.g., *E. coli *and *Methanococcus*) have only the NIF3 domains with the PII-like domain being absent. Experiments on spermatogonia derived cell line GC-1 suggest that nif3L1 (Ngg1-interacting factor 3-like 1) gene, a homolog of sa1388, expresses copiously during embryonic development and participates in retinoic acid-primed neural differentiation by interacting with the transcriptional corepressor Trip15/CSN2 [28]. Its primary role appears to be to inhibit Ngg1p from translocation to the nucleus, presumably by forming a binary complex in the cytoplasm [13]. Yeast two hybrid studies have shown that NIF3 binds to the amino terminal region of NGG1, an interaction that was implicated as a means of limiting transcriptional activation of GAL4p in glucose rich medium [30]. Furthermore, a genomewide two-hybrid analysis of yeast protein-protein interactions suggests that yeast NIF3p interacts with a nuclear import/export protein (Srp1p) and a ras-like GTPase (Temp1p), which are both required for proper exit from mitosis in the cell cycle [31]. In *E. coli*, expression of NIF3 homolog ybgI increases dramatically upon genotoxic stress induced by DNA damage [32]. This observation might be significant in light of the structure reported here because proteins that have been implicated in processes involving DNA metabolism often adopt toroidal structures [33-35]. However, the active site observed for SA1388 in this study, along with that seen in ybgI are not similar to the previously characterized DNA interacting toroids (e.g., the λ exonuclease [34]).

Although the precise biochemical function of the NIF3-like proteins are not known, the presence of a binuclear metal center similar to those found in hemerythrins, ribonucleotide reductases and purple acid phosphatase, as well as the endogenously bound ligand in the current structure strongly suggest that SA1388 NIF3 domains and *E. coli *Ybgl proteins likely bind and perform some form of catalysis on small molecule ligands, possibly metabolic substrates, and whose function may be regulated by the PII-like domains. In an attempt to determine the chemical identity of the endogenous ligand, we have extracted the ligand from the purified protein by trichloroacetic acid (TCA)/acetone precipitation. The preliminary high-resolution mass spectroscopic analysis of the TCA/acetone extract suggested that the ligand might be a compound of novel chemical composition. Crystallization of the compound has not been successful probably due to its intrinsic structural flexibility, which may not be susceptible for crystallization. Alternative approaches, such as NMR spectroscopy, may be needed in order to determine the chemical formulas and three-dimensional structure of the endogenous ligand.

## Conclusion

The recent upsurge in structures of a large number of hypothetical proteins by structural genomics efforts continue to provide important leads in the eventual determination of their function. This success has been largely due to either similarity of the overall structure, or local relatedness of catalytic residues to proteins of known function. Proteins with novel, previously unknown folds however are often limited in providing functional clues because of lack of similarity to any experimentally characterized proteins. Sometimes, the fortuitous observation of endogenously bound ligands offers important clues in this direction. The dinuclear metal center and the presence of a bound ligand at the active site of the NIF3 domain of SA1388 combined with the structural analysis of the two NIF3 homologs, ybgI and SA1609 should aid in the functional assignment of this widespread protein family.

## Methods

### Cloning, purification and crystallization

The gene coding for SA1388 was PCR amplified from genomic DNA of *Staphylococcus aureus *(ATCC, 700699D, Manassas, VA) and was cloned into a pProEX (Invitrogen) prokaryotic expression vector containing a trc promoter, 6xHis tag, and a tobacco etch virus (TEV) protease cleavage site. The resulting plasmid was transformed into the *E. coli *strain C41(DE3) (Imaxio, Clermont-Ferrand, France) for protein expression. Expression was induced at OD_600 _of 0.4 and the cells were pelleted after overnight growth at 20°C. Protein was first purified with a nickel-nitrilotriacetic acid (NTA)-agarose column (Qiagen) followed by cleavage of the N-terminal His tag by TEV protease. The protein was further purified by anion exchange chromatography on a MonoQ column (Amersham Biosciences) using a linear gradient of salt (Equilibration buffer: 50 mM Tris pH 7.8, 1 mM DTT; Elution buffer 50 mM Tris pH 7.8, 1 M NaCl, 1 mM DTT) and finally by gel-filtration chromatography on a Superdex 200 column (Amersham Biosciences) that was pre-equilibrated with a buffer containing 50 mM Tris pH 7.8, 300 mM NaCl, 1 mM DTT. The selenomethionine-substituted SA1388 protein was expressed in the minimum medium supplemented with selenomethionine and other nutrients according to standard protocols [36] and purified using the same procedure described above. Pure fractions of native and SeMet proteins were verified by SDS-PAGE and concentrated to 20 mg/ml for crystallization in a buffer containing 50 mM HEPES pH 7.2, 2 mM DTT and 250 mM NaCl. Crystals of SA1388 were grown by hanging drop vapour diffusion method. Single crystals of sizes around 0.3 × 0.3 × 0.2 mm^3 ^appeared within 3 days in the condition with 0.1 M Bis-Tris propane, pH 8.5, 0.2 M MgCl_2 _and 30% PEG 400 as the well solution. Crystals were directly frozen in liquid propane and stored in liquid nitrogen until data collection. All crystallographic data was processed using HKL2000 [37]

### Structure determination and refinement

Structure of SA1388 was solved by single wavelength anomalous dispersion (SAD) method. Initial phases were obtained from a complete 2.3 Å anomalous dataset collected at the peak wavelength of selenium at Advanced Photon Source (Argonne, IL), beamline 19-BM (SBC-CAT). Phasing using SOLVE [38] identified 16 (out of 22) selenium atoms with occupancies between 0.47–0.94 and figure of merit (FOM) of 0.31. Density modification with RESOLVE [39] assuming two molecules in the asymmetric unit (which gives Matthews' coefficient of 2.59 and solvent content of 53%) resulted in a clearly interpretable electron density map and an improved FOM of 0.67. Phases from RESOLVE were merged with a high resolution (2.0Å) native dataset using CAD of CCP4 suite followed by phase extension using DM [40] Automated model building program ARP-wARP [41] was able to build 63% of the protein residues including sidechains, mostly from the two NIF3 domains. The model was improved by alternating cycles of manual model building and refinement using Refmac5 of the CCP4 program suite [42,43]. The final statistics and refinement parameters are listed in Table 1. Structure factors and the coordinates have been deposited in the PDB (code 2NYD).

### Metal ion determination

X-ray fluorescence scans were performed at the Advanced Photon Source (APS) beam line 19-BM at Argonne National Laboratory on a native SA1388 crystal. Metal analysis was also conducted on a Perkin Elmer Optima 3300 DV Inductively Coupled Plasma (ICP) atomic emission spectrometer calibrated with AccuStandard ICP Multi-Element solutions. The protein was first dialyzed against a buffer containing 50 mM Hepes, pH7.5 and 1 mM DTT, and then diluted to appropriate concentrations for ICP atomic emission spectra analysis. The dialysis buffer was used as blank.

## Authors' contributions

KSS performed data collection, structure determination, structure-function analysis, and drafting of the manuscript. XZ cloned, expressed, purified and crystallized both native and SeMET forms of the protein. LK and NVG provided significant bioinformatics input and target selection in the project. ML performed ICP atomic emission and ICP mass spectrometry analysis. HZ coordinated all the components of the project, critically edited the manuscript, and provided financial support. All authors have read and approved the final manuscript.

## Figures and Tables

**Table 1:** Data collection and refinement statistics

**Data collection**		
**Crystal**	**SA1388_SeMET**	**SA1388_Native**

Wavelength (Å)	0.9792 (peak λ)	0.9792
Space group	H3	H3
Cell dimensions	*a *= *b *= 133.17 Å c = 125.80 Å	*a *= *b *= 132.54Å c = 125.67Å
Resolution range (Å)	50-2.3	50-2.0
Total number of observations	2275385	815894
Unique reflections	36981	55962
Completeness (%)	99.4 (100)	94.9(73.5)
Mean I/*σ*	14.23(2.98)	18.01(1.96)
*R*_merge_^1^	0.096 (0.529)	0.043(0.216)

**Refinement**		

Resolution range (Å)	502.0	
*R*_cryst_^2^	0.187	
*R*_free_^3^	0.229	
Bond length rmsd from ideal	0.018 Å	
Bond angle rmsd from ideal	1.78°	
Average isotropic *B*-value	35.1 Å^2^	
ESU based on free *R*	0.23 Å	

^1^R_merge _= ∑_hkl _∑_i_|I_i _(hkl) - <I(hkl)>|/∑_hkl _∑_i_I_i _(hkl)

^2^R_cryst _= ∑_hkl_||F_obs_| - k |F_calc_||/∑_hkl_|F_obs_|

^3 ^Five percent (total of 2675) of randomly chosen reflections was used to calculate R_free _Values in parenthesis correspond to the outer shell.

.
